# Supplementary material for: Applying particle filtering in both aggregated and age-structured population compartmental models of pre-vaccination measles
Source: PLoS One. 2018 Nov 2;13(11):e0206529. doi: 10.1371/journal.pone.0206529 (PMC6214536; doi:10.1371/journal.pone.0206529)
Supplement: S1 Appendix — (PDF) [file pone.0206529.s001.pdf]

S1 Appendix: The mathematical deduction of the age structured epidemiology model

Similar to the equilibrium demographic model in [1], the initial-boundary-value problem of the SEIR model with age structure could be listed as follows:

$$\begin{aligned}
&\frac{\partial S}{\partial t} + \frac{\partial S}{\partial a} = -\lambda(a,t)S - \mu(a)S \\
&\frac{\partial E}{\partial t} + \frac{\partial E}{\partial a} = \lambda(a,t)S - \sigma E - \mu(a)E \\
&\frac{\partial I}{\partial t} + \frac{\partial I}{\partial a} = \sigma E - \gamma I - \mu(a)I \\
&\frac{\partial R}{\partial t} + \frac{\partial R}{\partial a} = \gamma I - \mu(a)R \\
&\lambda(a,t) = \int_0^\infty \beta(a,a')\frac{I(a',t)}{N(a',t)}da'
\end{aligned} \tag{1}$$

where $\beta(a, a')$ is the infectious contact rate between age group a and age group a'.

The boundary condition at age 0 are all 0, except all the birth population going to $S(0, t)$:

$$S(0,t) = \int_0^\infty v(a)N(a,t)da \tag{2}$$

The initial conditions are the total population of each age group at time 0.

Similarly to the demographic model, we split the total population to n age groups. Then, for each age group i in the age interval $[a_{i-1}, a_i)$, we could get four ordinary differential equations of S_i, E_i, I_i, R_i. The definition of S_i, E_i, I_i, R_i are listed as follows:

$$\begin{aligned}
S_i(t) &= \int_{a_{i-1}}^{a_i} S(a,t)da \\
E_i(t) &= \int_{a_{i-1}}^{a_i} E(a,t)da \\
I_i(t) &= \int_{a_{i-1}}^{a_i} I(a,t)da \\
R_i(t) &= \int_{a_{i-1}}^{a_i} R(a,t)da
\end{aligned} \tag{3}$$

And for each age group i, we have:

$$N_i(t) = S_i(t) + E_i(t) + I_i(t) + R_i(t) \tag{4}$$

Thus, we could get totally $4n$ ordinary differential equations. The infectious contact rate $\beta(a, a')$ between any two age groups (a and a') are also assumed

to be constant. Then, we could have $\beta(a, a') = \beta_{ij}$, where age group i locates in the age interval $[a_{i-1}, a_i)$, and age group j locates in the age interval $[a_{j-1}, a_j)$. Similarly to the demographic model in [1], we have $S(a_i, t) = c_i S_i(t)$, $E(a_i, t) = c_i E_i(t)$, $I(a_i, t) = c_i I_i(t)$, $R(a_i, t) = c_i R_i(t)$. Finally, if we integrate the partial differential equations in the age interval $[a_{i-1}, a_i)$, we could get the final epidemiology model with $4n$ ordinary differential equations:

$$
\begin{aligned}
\frac{dS_1}{dt} &= \sum_{j=1}^{n} v_j N_j(t) - \lambda_1 S_1 - \mu_1 S_1 - c_1 S_1 \\
\frac{dE_1}{dt} &= \lambda_1 S_1 - \sigma_1 E_1 - \mu_1 E_1 - c_1 E_1 \\
\frac{dI_1}{dt} &= \sigma_1 E_1 - \gamma_1 I_1 - \mu_1 I_1 - c_1 I_1 \\
\frac{dR_1}{dt} &= \gamma_1 I_1 - \mu_1 R_1 - c_1 R_1 \\
\frac{dS_i}{dt} &= c_{i-1} S_{i-1} - \lambda_i S_i - \mu_i S_i - c_i S_i \quad i \geq 2 \\
\frac{dE_i}{dt} &= \lambda_i S_i + c_{i-1} E_{i-1} - \sigma_i E_i - \mu_i E_i - c_i E_i \quad i \geq 2 \\
\frac{dI_i}{dt} &= \sigma_i E_i + c_{i-1} I_{i-1} - \gamma_i I_i - \mu_i I_i - c_i I_i \quad i \geq 2 \\
\frac{dR_i}{dt} &= \gamma_i I_i + c_{i-1} R_{i-1} - \mu_i R_i - c_i R_i \quad i \geq 2 \\
\lambda_i &= \sum_{j=1}^{n} \beta_{ij} \frac{I_j}{N_j}
\end{aligned}
\tag{5}
$$

Specifically, the contact matrix in this age structured epidemiology model is: $\begin{bmatrix} \beta_{11} & \beta_{12} & \cdots & \beta_{1n} \\ \beta_{21} & \beta_{22} & \cdots & \beta_{2n} \\ \vdots & \vdots & \ddots & \vdots \\ \beta_{n1} & \beta_{n2} & \cdots & \beta_{nn} \end{bmatrix}$

References

[1] Hethcote HW. An age-structured model for pertussis transmission. Mathematical biosciences. 1997;145(2):89–136.
