# Supplementary material for: Applying particle filtering in both aggregated and age-structured population compartmental models of pre-vaccination measles
Source: PLoS One. 2018 Nov 2;13(11):e0206529. doi: 10.1371/journal.pone.0206529 (PMC6214536; doi:10.1371/journal.pone.0206529)
Supplement: S2 Appendix — (PDF) [file pone.0206529.s002.pdf]

S2 Appendix: The math deduction of the contact matrix model

Among the age groups, the number of contacts from the child age group to the adult age group in a given interval of time must equal the number of contacts from adults age group to the child age group in that same interval of time. This leads to the following equalities:

$$\begin{aligned} N_a C_a f_{ac} &= N_c C_c f_{ca} \\ f_{cc} + f_{ca} &= 1 \\ f_{ac} + f_{aa} &= 1 \end{aligned} \tag{1}$$

where C_a is the contact rate of the adults age group, while C_c is the contact rate of the child age group. Suppose the transmission probability is β_p, then we could have $\beta_c = C_c\beta_p$ and $\beta_a = C_a\beta_p$. It indicates that $\frac{C_c}{C_a} = \frac{\beta_c}{\beta_a}$.

Finally, by solving (1), we arrive at equation (2) below:

$$\begin{aligned} f_{ca} &= 1 - f_{cc} \\ f_{ac} &= \begin{cases} \frac{N_c\beta_c}{N_a\beta_a}\left(1 - f_{cc}\right), & \text{if } \left[\frac{N_c\beta_c}{N_a\beta_a}\left(1 - f_{cc}\right)\right] < 1.0 \\ 1.0, & \text{if } \left[\frac{N_c\beta_c}{N_a\beta_a}\left(1 - f_{cc}\right)\right] \geq 1.0 \end{cases} \\ f_{aa} &= 1 - f_{ac} \end{aligned} \tag{2}$$
