# Supplementary material for: Applying particle filtering in both aggregated and age-structured population compartmental models of pre-vaccination measles
Source: PLoS One. 2018 Nov 2;13(11):e0206529. doi: 10.1371/journal.pone.0206529 (PMC6214536; doi:10.1371/journal.pone.0206529)
Supplement: S3 Appendix — (PDF) [file pone.0206529.s003.pdf]

S3 Appendix: The complementary comments of the parameter of death rate

The death rate of the "infectious (I)" state should, in theory, be higher than the other states in the pre-vaccination era. According to measles history in CDC (Centers for Disease Control and Prevention) [1], there were 400 to 500 deaths reported among 3-4 million measles annually before 1963 (the vaccination starting year) in the United States. Thus, the measles causes death rate of the infectious state is about 0.125% to 0.167% yearly among the total population. Moreover, measles infection was nearly universal during childhood [1]. Thus, the measles attributable death rate of children should be higher than for adults. At the same time, the death rate among the total population of Saskatchewan during 1914 to 1921 is around 0.5% to 1.4%, for example, the death rate in 1921 of Saskatchewan is 0.81% [2]. However, the death rates of all states in the models of this paper are the same, to make the models simpler to be implemented.

References

[1] Measles History in Centers for Disease Control and Prevention.; 2018. Available from: https://www.cdc.gov/measles/about/history.html.

[2] Annual Report of Department of Public Health in the Province of Saskatchewan; 1921-1956.
