# Supplementary material for: Applying particle filtering in both aggregated and age-structured population compartmental models of pre-vaccination measles
Source: PLoS One. 2018 Nov 2;13(11):e0206529. doi: 10.1371/journal.pone.0206529 (PMC6214536; doi:10.1371/journal.pone.0206529)
Supplement: S4 Appendix — (PDF) [file pone.0206529.s004.pdf]

S4 Appendix: The mathematical deduction of the death rate

In this paper, we consider the population in each age group to be in equilibrium. This means the population of each age group stays same across the simulation. Thus, the demographic model should satisfy:

$$\begin{aligned} \frac{dN_c}{dt} &= N_a v_a - \mu_c N_c - \omega N_c = 0 \\ \frac{dN_a}{dt} &= \omega N_c - \mu_a N_a = 0 \end{aligned} \tag{1}$$

If we solve equation (1) for the death rates μ_a and μ_c, we could get the solutions of death rate of each age group. If the demographic model satisfies these solutions, this demographic model will remain in equilibrium. The solutions are:

$$\begin{aligned} \mu_c &= \frac{N_a}{N_c} v_a - \omega \\ \mu_a &= \frac{N_c}{N_a} \omega \end{aligned} \tag{2}$$

Equation (2) indicates that the value of death rate (μ_c, μ_a) is related to the fraction of population of the two age groups ($\frac{N_a}{N_c}$), the aging rate (ω), and the birth rate (v_a).
