# Supplementary material for: Applying particle filtering in both aggregated and age-structured population compartmental models of pre-vaccination measles
Source: PLoS One. 2018 Nov 2;13(11):e0206529. doi: 10.1371/journal.pone.0206529 (PMC6214536; doi:10.1371/journal.pone.0206529)
Supplement: S5 Appendix — (PDF) [file pone.0206529.s005.pdf]

S5 Appendix: The further introduction of the classification method

The method employed to address the binary classification problem is as follows: At time step *k-1*, we only calculate the state space model [1, 2] from time *k-1* to *k*, without the weight being updated by incorporating y_k. As a result, the weight of each particle is the prior one calculated in time step *k-1*. Hence, the state vector $x_k^{N(i)}$ with the weight $w_{k-1}{}^{(i)}$ represents the prediction results for time step *k*, on the basis only of data available until time *k-1*. As a result, we could get the predicted reported measles cases of particles generated by importance sampling of the particles – that is, sampling those particles according to weight. Following this, all the $I_{\mathrm{rpk}}^{(i)}$ will make up a matrix. One dimension is the time steps (k). The other dimension is the particles (i).

To perform the classification analysis, we need to conduct two processes. In the first process, we define the threshold of a particle i at time step k above which to consider that particle as positing an outbreak (θ_{ik}). In this project, we suppose that the reported count threshold above which we consider there as being an outbreak is "mean plus 1.5 times the standard deviation of the empirical monthly reported cases". As given by equation (1) and (2), if for a particle i we have $I_{\mathrm{rpk}}^{(i)} \geq \theta_{\mathrm{ik}}$, we label this data $I_{\mathrm{rpk}}^{(i)}$ as an "outbreak". Otherwise, it is considered not to be an outbreak. In the second process, we define a threshold of fraction (θ_k) of particles required to posit an outbreak at time k for us to consider there as being an outbreak at time *k*. The equations for classification are listed as follows:

$$z_{\mathrm{ik}} = \begin{cases} 1, I_{\mathrm{rpk}}^{(i)} \geq \mu^E + 1.5\sigma^E \\ 0, I_{\mathrm{rpk}}^{(i)} < \mu^E + 1.5\sigma^E \end{cases} \tag{1}$$

$$z_k = \begin{cases} 1, \sum_{i=1}^{N_s} z_{\mathrm{ik}} \geq \ \theta_k * N_s \\ 0, \sum_{i=1}^{N_s} z_{\mathrm{ik}} < \ \theta_k * N_s \end{cases} \quad (0 \leq \theta_k \leq 1) \tag{2}$$

Where μ^E is the mean of empirical data, σ^E is the standard deviation of the empirical data, and N_s is the total number of particles.

We could get the predicted classification vector $\{z_k\}_{k=1}^{T_f}$ by conducting the particle filtering algorithm and applying equation (1) and (2). We further denote $\{y_{lk}\}_{k=1}^{T_f}$ as the empirical vector of whether a measles outbreak indeed obtained at time *k*, $y_{lk} \in \{0, 1\}$. The calculation method of y_{lk} is similar to that of z_{ik}:

$$y_{lk} = \begin{cases} 1, y_k^M \geq \mu^E + 1.5\sigma^E \\ 0, y_k^M < \mu^E + 1.5\sigma^E \end{cases} \tag{3}$$

Where y_k^M is the empirical data of measles reported cases.

References

[1] Murphy KP. Machine Learning: A Probabilistic Perspective. Cambridge, MA: The MIT Press; 2012.

[2] Arulampalam MS, Maskell S, Gordon N, Clapp T. A tutorial on particle filters for online nonlinear/non-Gaussian Bayesian tracking. IEEE Transactions on signal processing. 2002;50(2):174–188.
