# Supplementary material for: Applying particle filtering in both aggregated and age-structured population compartmental models of pre-vaccination measles
Source: PLoS One. 2018 Nov 2;13(11):e0206529. doi: 10.1371/journal.pone.0206529 (PMC6214536; doi:10.1371/journal.pone.0206529)
Supplement: S6 Appendix — (PDF) [file pone.0206529.s006.pdf]

S6 Appendix: The shortcoming of choosing the binomial distribution as the likelihood function

One way of characterizing the reporting process of measles would be analogous to a coin flip, with each case being subject to an independent distributed probability of reporting. Such a treatment of reporting would imply a likelihood function characterized by the probability mass function of a binomial distribution, with a count of trials equal to the count of underlying incident cases posited by a particle.

However, if we choose a binomial distribution as the basis for a likelihood function in this project, it imposes a high risk of causing a problem of singularity during weight re-normalization [1]. This can be caused by situations where all particles in the model are associated with an infectious state smaller than the empirical data observed. Because the number of trials for each such particles will be less than the value of the empirical incident case count, the probability of a binomial draw yielding the observed data is 0 for each particle and thus the weights of each particle would become zero.

References

[1] Osgood N, Liu J. Towards closed loop modeling: Evaluating the prospects for creating recurrently regrounded aggregate simulation models using particle filtering. In: Simulation Conference (WSC), 2014 Winter. IEEE; 2014. p. 829–841.
