# Supplementary material for: Applying particle filtering in both aggregated and age-structured population compartmental models of pre-vaccination measles
Source: PLoS One. 2018 Nov 2;13(11):e0206529. doi: 10.1371/journal.pone.0206529 (PMC6214536; doi:10.1371/journal.pone.0206529)
Supplement: S7 Appendix — (PDF) [file pone.0206529.s007.pdf]

S7 Appendix: The further introduction of split the measles yearly reported cases to each age group.

The yearly empirical data related to multiple age categories are available from year 1925 to 1956. During the process in preparing the yearly empirical data match the two age groups in the two age group particle filtering models (children age group is for those people up to 5 or up to 15 years), we need to split some age categories due to two reasons. The first reason is because the division of the age group in empirical dataset does not match the two age groups in particle filtering models. Specifically, from year 1926 to 1941, we need to split the counts of reported measles cases in age category "1-6 years" in age 5 proportionally (four fifths goes to the child age group, and one fifth goes to adult age group). This problem only related to the age group model of child group up to 5 years old. The second reason is because there is a category in the empirical yearly dataset of "age not stated". Thus, we need to split the counts in this category to corresponding age groups with the two age group models – the persons in the child age group up to 5 or 15 years old proportionally (based on the proportion calculated by the age categories has labeled age clearly).
