# Supplementary material for: Applying particle filtering in both aggregated and age-structured population compartmental models of pre-vaccination measles
Source: PLoS One. 2018 Nov 2;13(11):e0206529. doi: 10.1371/journal.pone.0206529 (PMC6214536; doi:10.1371/journal.pone.0206529)
Supplement: S1 Table — (PDF) [file pone.0206529.s008.pdf]

S1 Table: The initial values of all models

Table 1: Table Showing Initial Values of The S, E, I, R Stocks in The Particle Filtering Models. The Units Are All Person.

**Parameter**	**Value**
S_0	96354 - I_0
E_0	0
I_0	Uniform[0, 9635)
R_0	767191
S_{0c5}	43177 - I_{0c5}
E_{0c5}	0
I_{0c5}	Uniform[0, 4318)
R_{0c5}	55566
S_{0a5}	43177 - I_{0a5}
E_{0a5}	0
I_{0a5}	Uniform[0, 4318)
R_{0a5}	721625
S_{0c15}	67454 - I_{0c15}
E_{0c15}	0
I_{0c15}	Uniform[0, 6745)
R_{0c15}	219083
S_{0a15}	28900 - I_{0a15}
E_{0a15}	0
I_{0a15}	Uniform[0, 2890)
R_{0a15}	548108
